# A General Process-Based Model for Describing the Metabolic Shift in Microbial Cell Cultures

**DOI:** 10.3389/fmicb.2020.521368

**Published:** 2020-09-30

**Authors:** Fabrizio Carteni, Alessio Occhicone, Francesco Giannino, Christian E. Vincenot, Elisabetta de Alteriis, Emanuela Palomba, Stefano Mazzoleni

**Affiliations:** ^1^Department of Agricultural Sciences, University of Naples Federico II, Portici, Italy; ^2^Department of Engineering, University of Naples Parthenope, Naples, Italy; ^3^Department of Social Informatics, Kyoto University, Kyoto, Japan; ^4^Department of Biology, University of Naples Federico II, Naples, Italy; ^5^Section Research Infrastructure for Marine Biological Resources, Stazione Zoologica Anton Dohrn, Naples, Italy

**Keywords:** *Escherichia coli*, *Bacillus subtilis*, System Dynamics (SD) model, self-inhibition, high cell-density culture, overflow metabolism, Crabtree/Warburg effect

## Abstract

The metabolic shift between respiration and fermentation at high glucose concentration is a widespread phenomenon in microbial world, and it is relevant for the biotechnological exploitation of microbial cell factories, affecting the achievement of high-cell-densities in bioreactors. Starting from a model already developed for the yeast *Saccharomyces cerevisiae*, based on the System Dynamics approach, a general process-based model for two prokaryotic species of biotechnological interest, such as *Escherichia coli* and *Bacillus subtilis*, is proposed. The model is based on the main assumption that glycolytic intermediates act as central catabolic hub regulating the shift between respiratory and fermentative pathways. Furthermore, the description of a mixed fermentation with secondary by-products, characteristic of bacterial metabolism, is explicitly considered. The model also represents the inhibitory effect on growth and metabolism of self-produced toxic compounds relevant in assessing the late phases of high-cell density culture. Model simulations reproduced data from experiments reported in the literature with different strains of non-recombinant and recombinant *E. coli* and *B. subtilis* cultured in both batch and fed-batch reactors. The proposed model, based on simple biological assumptions, is able to describe the main dynamics of two microbial species of relevant biotechnological interest. It demonstrates that a reductionist System Dynamics approach to formulate simplified macro-kinetic models can provide a robust representation of cell growth and accumulation in the medium of fermentation by-products.

## Introduction

Glucose is the main carbon and energy source for microbial metabolism. Glucose uptake supplies the glycolytic process producing different intermediates, with pyruvate representing a central catabolic hub, followed by the respiratory or fermentative pathway, depending on oxygen availability.

Respiration is able to maximize ATP production and consequently biomass yield. However, despite the fully aerobic conditions, in several microbial species when glucose concentration is high, the respiratory metabolism is replaced by a fermentative one, which produces partially oxidized products (Molenaar et al., 2009; Goel et al., 2012).

Such metabolic shift between two different ATP producing metabolisms, respiration and fermentation, is a widespread phenomenon in the biological world (Molenaar et al., 2009; Goel et al., 2012). In yeast it is known as Crabtree effect (De Deken, 1966) recognizing its similarity with the respiration/fermentation shift occurring in mammalian cells where it is commonly reported as Warburg effect (Warburg, 1956), and considered a hallmark of cancer (Hanahan and Weinberg, 2011). Moreover, some yeasts are recognized to be Crabtree-positive such as *S. cerevisiae*, others are Crabtree-negative (De Deken, 1966), the difference mainly relies on the extent of the glycolytic flux which, in turn, depends on the glucose uptake rate (Huberts et al., 2012). Recently, it has been shown how the overexpression of a single transcription factor (the ortholog of *S. cerevisiae GAL4*) in *Komagataella phaffii* results in a switch of the Crabtree phenotype from negative to positive with an increase in specific glucose uptake (Ata et al., 2018).

The fitness advantage associated to the metabolic shift and, more in general its significance, has been largely debated (Pfeiffer and Morley, 2014; Liberti and Locasale, 2016). Recently, a review and clarification of the process dynamics beyond this phenomenon has been proposed (de Alteriis et al., 2018).

The metabolic shift has been attributed to an “overflow metabolism,” caused by the saturation of the limited respiratory capacity of the cell, leading to an overflow reaction at pyruvate level, as first shown for the yeast *Saccharomyces cerevisiae* (Sonnleitner and Käppeli, 1986). It is now established that a complex interplay of molecular mechanisms is also responsible for the phenomenon, with the ascertained role of regulatory systems referred to as either carbon catabolite or glucose repression in prokaryotes (Sonenshein, 2007; Bernal et al., 2016) and yeast (Westergaard et al., 2007), respectively.

As known, the prokaryotic cell factories *Escherichia coli*, and to a lesser extent *Bacillus subtilis*, together with the eukaryotic unicellular fungus *Saccharomyces cerevisiae* are the prevalent microbial platforms for biotechnological applications (Öztürk et al., 2016; Sanchez-Garcia et al., 2016). The phenomenon of the metabolic shift with the consequent production of fermentative products has been widely described for these species, representing one of the problems which may limit the achievement of high cell densities and productivities (Riesenberg et al., 1991; Sandén et al., 2003), for both non-recombinant and recombinant microbial strains (Lee, 1996; Riesenberg and Guthke, 1999; Porro et al., 2005; Shiloach and Fass, 2005; Öztürk et al., 2016).

In unrestricted growth conditions, the fermentative microbial metabolism generally leads to a main end-product and other by-products: *E. coli* and *B. subtilis* predominantly form acetate, but also lactate and propionate respectively, while in the case of *S. cerevisiae* a production of ethanol and, to lesser extent, acetate is observed.

In the case of *E. coli*, aerobic acetate production is very detrimental for growth and productivity, and for this reason it has been largely investigated (Wolfe, 2005; De Mey et al., 2007; Bernal et al., 2016). Several strategies have been proposed to avoid acetate production, from technological approaches (fed-batch cultures with controlled glucose supply, removal of acetate from culture medium, use of alternative carbon sources such as glycerol or mannose) to genetic approaches aimed to obtain strains with low propensity to acetate formation (Sandén et al., 2003; Shiloach and Fass, 2005; Eiteman and Altman, 2006).

The outstanding importance of *E. coli* in biotechnological processes supported the development of several mathematical models aimed to describe strain performance in different cultural conditions, optimizing their cell/product density and avoiding acetate overproduction. First attempts to use mechanistic models to simulate the kinetics of *E. coli* population growth followed different approaches, from simplified representations of batch cultures (Corman et al., 1986), based on classic biomass-resource model (Monod, 1949) to more detailed models of the main metabolic fluxes by an optimization method (Majewski and Domach, 1990; Ko et al., 1993, 1994). More recently, a process-based kinetic model first developed by Xu et al. (1999) was further improved to study the growth of *E. coli* W3110 strain in batch and fed-batch cultures, explicitly including the inhibitory effect of acetate accumulation on glucose and oxygen consumption (Lin et al., 2001; Neubauer et al., 2003).

The recent increasing studies on *E. coli* metabolism improved the understanding of the acetate production on one hand and, on the other hand, the co-assimilation of both acetate and glucose in sugar-limited conditions (Wolfe, 2005; Lara et al., 2008; Peebo et al., 2015; Basan et al., 2015; Bernal et al., 2016). Such new findings were integrated in new macro-kinetic models (Anane et al., 2017; Retamal et al., 2018). In particular, Anane et al. (2017) developed a mechanistic model based on previous works (Xu et al., 1999; Lin et al., 2001; Neubauer et al., 2003) with two major improvements: (i) a mathematical formulation deriving a set of tractable and continuously differentiable equations leading to better computational performance and allowing the use of gradient-based optimization methods and (ii) the inclusion of a continuous process of production and re-assimilation of intracellular acetate even under non-overflow conditions, as recently highlighted in proteomic and systems biology studies (Valgepea et al., 2010; Basan et al., 2015; Peebo et al., 2015). The model proposed by Retamal et al. (2018), based on the overflow metabolism assumption (Sonnleitner and Käppeli, 1986), assumed that the critical glucose uptake rate responsible for the activation of the metabolic overflow is not constant, but decreases with increasing acetate concentrations.

Following the consideration that the metabolic shift in *S. cerevisiae* is controlled by both limited respiratory capacity (Sonnleitner and Käppeli, 1986) and repression of respiration (Westergaard et al., 2007), our group developed a novel macro-kinetic model based on the System Dynamics approach (Forrester, 1961), capable to reproduce the growth of the budding yeast in both batch and high-cell-density cultures (Mazzoleni et al., 2015). The main assumption of this model was that the glycolytic intermediates represent the central metabolic hub regulating the shift between respiratory and fermentative pathways.

In this work, considering the similarity of the metabolic shift between *S. cerevisiae* and prokaryotic cells as determined by the level of the glycolytic intermediates, we extend the model by Mazzoleni et al. (2015) to simulate the growth of different strains of *E. coli* and *B. subtilis* cultured in batch and fed-batch bioreactors under aerobic conditions, with glucose as carbon and energy source.

## Materials and methods

**FIGURE 1:**
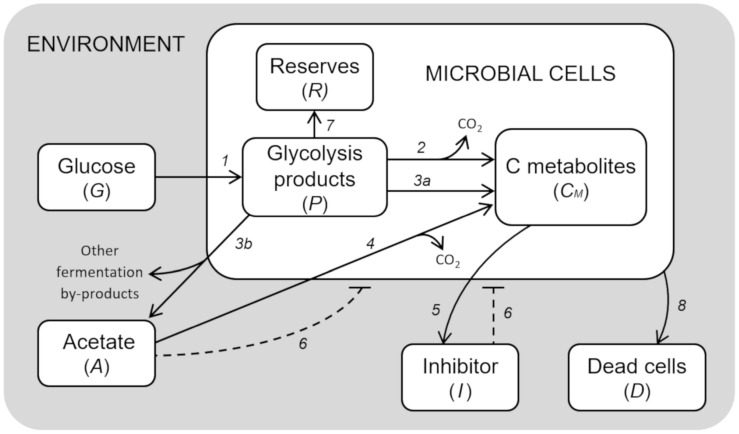
Model diagram of microbial growth. Simplified cell metabolism with explicit representation of the major metabolic pathways. (1) Glucose uptake; (2) respiration; (3a) fermentation; (3b) acetate production by mixed fermentation; (4) acetate respiration; (5) secretion of inhibitory compounds; (6) inhibitory effects; (7) reserves accumulation; (8) cell death.

The model developed to simulate the growth behavior of a generic microbial cell cultured in a bioreactor is presented. Figure 1 shows a schematic diagram of the implemented processes, the limited number of which was achieved by a top-down approach, i.e., selecting the essential elements sufficient to reach a robust representation of the system behavior.

The resulting model is composed of a set of 7 ordinary differential equations representing glucose in the growth medium (*G*), glycolysis intermediates from glucose-6-phosphate to pyruvate (*P*), acetate produced by fermentation (*A*), cellular components produced by either fermentation or respiration (*C*_*M*_), reserve compounds (*R*), growth-associated inhibitory by-products (*I*), and dead cells (*D*).

Glucose (*G*) is provided according to the feeding conditions of the bioreactor described for the simulated experiment. Glucose is assimilated by microbial cells, and then converted into the different intermediate products of glycolysis, from glucose-6-phosphate to pyruvate (*P*). These are used for the construction of new cellular material (*C*_*M*_), either through respiration or fermentation. In the case of fermentation, acetate (*A*) is the main end-product, which can also be used as carbon source for the respiratory pathway when glucose is limiting.

The essential assumption of the model is the key role of the glycolytic products (*P*) in the regulation of the metabolic shift between respiration/fermentation and, in general, cell metabolism. Therefore, high levels of *P* are assumed to be responsible for (i) the activation of aerobic fermentation due to overflow metabolism, (ii) the repression of respiration (“glucose effect”), (iii) the accumulation of reserve materials (*R*), and (iv) the induction of mortality with accumulation of dead cells (*D*) (de Alteriis et al., 2018).

Moreover, considering that acetate is not the only end-product of bacterial fermentations even in aerobic conditions (Park et al., 1992; Kim et al., 2015), in the presented model we also assumed that the allocation toward secondary by-products proceeds in parallel with acetate. In particular, their production is assumed to increase with the acetate outflow from the cells.

The model also considers growth-associated inhibitory by-products (*I*), as already described in Mazzoleni et al. (2015), whose production is related to anabolic pathways, hence it is expressed as a proportion of the respiration and fermentation fluxes. Both the inhibitors and acetate are assumed to separately exert a negative feedback on cell growth in a concentration-dependent way.

The model is formulated with the following mass-balance equations:

$$\frac{d\,G}{d\,t}=F\,e\,e\,d\,i\,n\,g-U\,p\,t\,a\,k\,e$$

$$\frac{d\,P}{d\,t}=\eta_{G}\cdot U\,p\,t\,a\,k\,e-R\,e\,s\,p\,i\,r\,a\,t\,i\,o\,n_{P}-F\,e\,r\,m\,e\,n\,t\,a\,t\,i\,o\,n-A\,c\,c\,u\,m\,u\,l\,a\,t\,i\,o\,n-D\,e\,a\,t\,h_{P}$$

$$\frac{d\,A}{d\,t}=\eta_{F\,A}\cdot F\,e\,r\,m\,e\,n\,t\,a\,t\,i\,o\,n\cdot f\,a-R\,e\,s\,p\,i\,r\,a\,t\,i\,o\,n_{A}$$

$$\frac{d\,C_{M}}{d\,t}=\eta_{R\,P}\cdot R\,e\,s\,p\,i\,r\,a\,t\,i\,o\,n_{P}+\eta_{R\,A}\cdot R\,e\,s\,p\,i\,r\,a\,t\,i\,o\,n_{A}+\eta_{F\,P}\cdot F\,e\,r\,m\,e\,n\,t\,a\,t\,i\,o\,n-S\,e\,c\,r\,e\,t\,i\,o\,n-D\,e\,a\,t\,h_{C\,M}$$

$$\frac{d\,R}{d\,t}=\eta_{A}\cdot A\,c\,c\,u\,m\,u\,l\,a\,t\,i\,o\,n-D\,e\,a\,t\,h_{R}$$

$$\frac{d\,I}{d\,t}=S\,e\,c\,r\,e\,t\,i\,o\,n$$

$$\frac{d\,D}{d\,t}=D\,e\,a\,t\,h_{P}+D\,e\,a\,t\,h_{C\,M}+D\,e\,a\,t\,h_{R}$$

The equations of the model are described in detail in Tables 1, 2, while fixed and calibrated parameters are described in Tables 3, 4.

**TABLE 1:** Model processes.

**Equation**
$Feeding\;=\;\{\begin{array}{cc} C_{F}\;\cdot \;F_{0}\;\cdot \;\exp(\mu (t\;-\;t_{F})), & exponential\;feeding\\ C_{F}F_{0},\; & linear\;feeding \end{array}$
$U\,p\,t\,a\,k\,e=v_{G}\,\frac{\left[G\right]}{k_{G}+\left[G\right]}\,B\,\left(1-\frac{\left[P\right]}{{\left[P\right]}_{m\,a\,x}}\right)\,\left(1-n_{E}\right)\,l\,a\,g$
$R\,e\,s\,p\,i\,r\,a\,t\,i\,o\,n_{P}=v_{R\,P}\,\frac{\left[P\right]}{k_{R\,P}+\left[P\right]}\,B\,\left(1-n_{A}\right)\,\left(1-n_{I}\right)\,g\,e$
$F\,e\,r\,m\,e\,n\,t\,a\,t\,i\,o\,n=v_{F}\,\frac{\left[P\right]}{k_{F}+\left[P\right]}\,B\,\left(1-n_{A}\right)\,\left(1-n_{I}\right)\,m\,o$
$R\,e\,s\,p\,i\,r\,a\,t\,i\,o\,n_{A}=v_{R\,A}\,\frac{\left[A\right]}{k_{R\,A}+\left[A\right]}\,B\,\left(1-n_{A}\right)\,\left(1-n_{I}\right)\,g\,e$
$A\,c\,c\,u\,m\,u\,l\,a\,t\,i\,o\,n=v_{A}\,\frac{\left[P\right]}{k_{A}+\left[P\right]}\,B\,\left(1-\frac{R}{R_{m\,a\,x}}\right)\,m\,o$
*Secretion* = ρ(*η*_*RP*_⋅*Respiration*_*P*_ + *η*_*RA*_⋅*Respiration*_*A*_ + *η*_*FP*_⋅*Fermentation*)
*Death*_*P*_ = *d*⋅*δ*⋅*P*
*Death*_*R*_ = *d*⋅*δ*⋅*R*
*Death*_*CM*_ = *d*⋅*δ*⋅*C*_*M*_

*All symbols are described in Tables 2–4.*

**TABLE 2:** Symbols used in the model equations.

**Description**	**Formula**
Initial feed rate	$F_{0}=\left\{ \begin{array}{cc} 0, & t<t_{F}\\ \frac{M_{F}\cdot \mu }{c_{F}\cdot y_{R}}, & t\geq t_{F} \end{array}\right.$
Glucose concentration	$\left[G\right]=\frac{G}{V}$
Active metabolite mass	*B = P+C_*M*_*
Glycolysis products concentration	$\left[P\right]=\frac{P}{\left(B+R\right)\,c}$
Acetate negative feedback	$n_{A}=\sigma_{A}\,\frac{\left[A\right]}{{\left[A\right]}_{m\,a\,x}}$
Lag phase	$lag\;=\;\{\begin{array}{cc} 0, & t\;<\;t_{L}\\ 1, & t\;\geq \;t_{L} \end{array}$
Inhibitor negative feedback	$n_{I}=\sigma_{I}\,\frac{\left[I\right]}{{\left[I\right]}_{m\,a\,x}}$
Glucose effect	$g\,e=\frac{1}{1+a_{1}\cdot \text{exp}\,\left(b_{1}\cdot \left[P\right]\right)}$
Metabolic overflow	*mo* = 1−*ge*
Fermentation allocation to acetate	$f\,a=\frac{1}{1+a_{2}\cdot \text{exp}\,\left(b_{2}\cdot \left[A\right]\right)}$
Acetate concentration	$\left[A\right]=\frac{A}{V}$
Maximum reserves	*R*_*max*_ = *(B+R) r_MAX_*
Death switch	$d\;=\;\{\begin{array}{cc} 0, & [P]\;\leq \;\tau \\ 1, & [P]\;>\;\tau \end{array}$
Medium volume in the reactor	$V\,\left(t\right)=\left\{ \begin{array}{cc} V_{0}, & t<t_{F}\\ V_{0}+\frac{M_{F}}{c_{F}\cdot y_{R}}\,\text{exp}\,\left(\mu \cdot \left(t-t_{F}\right)\right), & t_{F}\leq t\leq t_{E\,N\,D} \end{array}\right.$

*Other parameters are described in Tables 3, 4.*

**TABLE 3:** State variables initial values and simulation setup parameters.

**Symbol**	**Description**	**Unit**	**Figure 2*E. coli*W3110** (Anane et al., 2017)	**Figure 3*E. coli*TG1** (Riesenberg et al., 1991)	**Figure 3*E. Coli*TG1** (Korz et al., 1995)	**Figure 4*E. coli* TG1 recombinant** (Hellmuth et al., 1994)	**Figure 4*E. coli* TG1 recombinant** (Rinas and Hoffmann, 2004)	**Figure 5*B. subtilis*** (Huang et al., 2004)
*G*_0_	Glucose initial value	g	5	25	28.5	27.75	25	4.4
*A*_0_	Acetate initial value	g	0	0	0	0	0	0
*P*_0_	Glycolysis products initial value	g	5 ⋅ 10^–5^	5 ⋅ 10^–5^	5 ⋅ 10^–5^	5 ⋅ 10^–5^	5 ⋅ 10^–5^	5 ⋅ 10^–5^
*C*_*M0*_	Carbon metabolites initial value	g	0.2	0.1	0.1	0.1	0.1	0.01
*I*_0_	Inhibitor initial value	g	0	0	0	0	0	0
*R*_0_	Reserve compounds initial value	g	0	0	0	0	0	0
*D*_0_	Dead cells initial value	g	0	0	0	0	0	0
*t*_0_	Time of simulation start	h	0	0	0	0	0	0
*t*_*F*_	Time of exponential feeding start	h	11.5	12	9; 11	13	16.5	10.5
*t*_*Flin*_	Time of linear feeding start	h	16	30	–	–	–	–
*t*_*END*_	Time of simulation end	H	33	35	22	24	31	27.3
*c*_*F*_	Glucose concentration in feeding solution	g l^−1^	500	770	500	500	500	500
*M*_*F*_	Cell mass at beginning of feeding	g	4.6; 20.5	10; 94	20; 25	13	11	1.85
*μ*	Feeding rate	h^–1^	0.3; 0.19	0.11; 0.08	0.17; 0.14	0.12	0.13	0.24
*y*_*R*_	Maximum biomass yield on glucose	–	0.39	0.42	0.5	0.5	0.5	0.39
*lag*	Lag phase calibration parameter	h	–	4	–	5	7.5	–

*Each column heading reports: the article figure showing the simulation, the microbial species/strain, and the publication reference (indicated in the square brackets) of the different experimental settings.*

**TABLE 4:** Model calibrated parameters with description and simulation values for each microbial strain.

**Symbol**	**Description**	**Unit**	***E. coli*W3110**	***E. coli*TG1**	***E. coli* TG1 recombinant**	***B. subtilis***
*v*_*G*_	Maximum uptake rate	h^–1^	0.82	1.92	1.92	1.98
*k*_*G*_	Uptake saturation constant	g l^−1^	0.005	2.02	2.02	0.15
*η_*G*_*	Uptake efficiency *P*/*G*	–	0.69	0.91	0.91	0.65
*v*_*RP*_	Maximum glycolysis products respiration rate	h^–1^	0.09	0.72	0.72	0.23
*k*_*RP*_	Glycolysis products respiration sat constant	g l^−1^	0.02	0.02	0.02	0.01
*η_*RP*_*	Respiration efficiency *C*_*M*_/*P*	–	0.76	0.71	0.71	0.09
*v*_*F*_	Maximum fermentation rate	h^–1^	0.46	5.57	5.57	1.08
*k*_*F*_	Fermentation saturation constant	g l^−1^	0.08	0.01	0.01	0.04
*η_*FA*_*	Fermentation efficiency *A*/*P*	–	0.12	0.37	0.37	0.99
*η_*FP*_*	Fermentation efficiency *C*_*M*_/*P*	–	0.94	0.31	0.31	0.92
*v*_*RA*_	Maximum acetate respiration rate	h^–1^	0.25	0.04	0.04	0.35
*k*_*RA*_	Acetate respiration saturation constant	g l^−1^	0.86	0.12	0.12	0.21
*η_*RA*_*	Respiration efficiency *C*_*M*_/*A*	–	0.94	0.43	0.43	0.96
*v*_*A*_	Maximum accumulation rate	h^–1^	0.2	0.2	0.2	0.2
*k*_*A*_	Accumulation saturation constant	g l^−1^	0.05	0.05	0.05	0.05
*η_*A*_*	Accumulation efficiency *R*/*P*	–	0.93	0.45	0.45	0.99
*r*_MAX_	Maximum reserves / cell mass ratio	–	0.3	0.3	0.3	0.3
*δ*	Death rate	h^–1^	0.1	0.1	0.1	0.1
*τ*	Death threshold	g l^−1^	0.6	0.6	0.6	0.6
ρ	Secretion rate	h^–1^	0.003	0.008	0.008	0.052
*σ_*I*_*	Sensitivity to inhibitor NF	–	1.0	1.0	1.0	1.0
*σ_*A*_*	Sensitivity to acetate NF	–	2.71	1.23	1.23	1.15
*c*	Cell volume/dry weight ratio	l g^−1^	0.01	0.01	0.01	0.01
*a*_1_	Resp/ferm metabolic switch	–	3.0 ⋅ 10^–4^	3.4 ⋅ 10^–4^	3.4 ⋅ 10^–4^	1.6 ⋅ 10^–4^
*b*_1_	Resp/ferm metabolic switch	l g^−1^	35.5	34.1	34.1	23.4
*a*_2_	Mixed fermentation metabolic switch	–	0.051	0.015	0.015	0.010
*b*_2_	Mixed fermentation metabolic switch	l g^−1^	4.9	7.6	15.6	21.0

The mathematical equations were integrated using MATLAB R2018b (the MathWorks) with a variable order solver (ode15s). The model calibration was performed by minimizing the sum of the squared errors (SSE)

$$S\,S\,E=\frac{1}{n_{1}}\,\sum_{i=1}^{n_{1}}{\left(C_{M\,i}-C_{M\,i}^{*}\right)}^{2}+\frac{1}{n_{2}}\,\sum_{i=1}^{n_{2}}{\left(G_{i}-G_{i}^{*}\right)}^{2}+\frac{1}{n_{3}}\,\sum_{i=1}^{n_{3}}{\left(A_{i}-A_{i}^{*}\right)}^{2}$$

where n_1_, n_2_, n_3_ are the number of samples per observed outputs, *C*_*Mi*_, *G_i*, *A_i*, are the values of the *i*th measured outputs and $C_{M\,i}^{*}$, $G_{i}^{*}$, $A_{i}^{*}$, are the values of the *i*th outputs predicted by the model. The minimization was performed by using the fminsearch MATLAB routine which implements a Nelder–Mead simplex algorithm (Lagarias et al., 1998).

Furthermore, a sensitivity analysis was implemented to analyze the model behavior under parameters perturbations. Using a local sensitivity analysis (Morris, 1991; Norton, 2015), the following normalized sensitivity index was calculated by changing each parameter by ± 5% one-at-a time while keeping the rest constant:

$$S\,S\,E_{i,△}=$$

$$=\sqrt{\frac{1}{n_{1}}\,\sum_{j=1}^{3}\sum_{i=1}^{n_{1}}{\left(\frac{X^{j}\,\left(p_{1},p_{2},\ldots ,p_{i}+△,\ldots ,p_{k}\right)-X^{j}\,\left(p\right)}{m\,a\,x\,\left(X^{j}\,\left(p\right)\right)-m\,i\,n\,\left(X^{j}\,\left(p\right)\right)}\right)}^{2}}$$

where, *SSE*_*i*,△_ is the Standardized elementary effect of the parameter *p*_*i*_ with △ (± 5%) perturbation on the model outputs; *X*^*j*^(***P***) represents the simulation values of the state variables Microbial mass, Glucose and Acetate without any parameter perturbation; *max*(*X*^*j*^(**p**))−*min*(*X*^*j*^(**p**)) is the standardization factor referring to the values of the baseline simulation; *k* is the number of parameters.

## Results

Model simulations were compared to experiments of growth in bioreactor of two strains of *E. coli* among the most used in biotechnological applications, namely W3110 (Anane et al., 2017) and TG1 (Riesenberg et al., 1991; Korz et al., 1995). For *E. coli* TG1, also experiments of two recombinant strains were considered (Hellmuth et al., 1994; Rinas and Hoffmann, 2004). In the case of *B. subtilis*, model simulation was compared to the experiment by Huang and co-workers (Huang et al., 2004).

In the experiments selected for model simulations, microbial growth was carried out in bioreactors initially operating in batch mode and then fed with a glucose-based inlet stream (fed-batch). The possible metabolic shift occurred according to the value of the specific growth rate of the population, determined by the specific feeding rate (SFR) applied to the bioreactor during the fed-batch phase (Enfors and Häggström, 1998).

**FIGURE 2:**
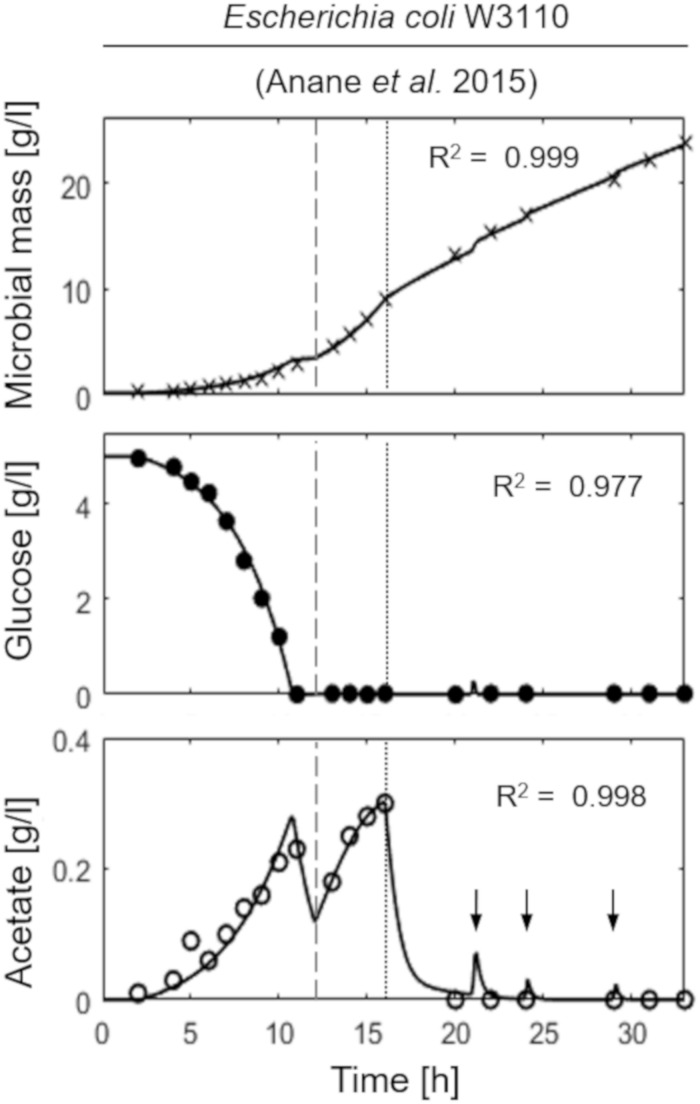
Measured vs. simulated growth of *Escherichia coli* W3110 reproducing the experiment in Anane et al. (2017). Time series of measured microbial mass (*times symbol*), glucose (*filled circle*), and acetate (*open circle*) data vs. model simulations (*continuous lines*). *Dashed vertical lines* represent the beginning of exponential feeding, while *dotted vertical lines* the beginning of constant feeding (arrows indicate glucose pulses).

Figure 2 shows the model simulation reproducing a two-phase (exponential and constant feeding regimes) fed-batch culture of *E. coli* W3110, performed by Anane et al. (2017). The initial batch culture, characterized by a maximum specific growth rate value (μ_MAX_) of 0.31 h^–1^, presents the typical exponential growth behavior of an *E. coli* population growing on glucose and displaying a fermentative metabolism with acetate production. When glucose is completely depleted in the medium, fermentative metabolism is replaced by a respiratory one, with a short period of acetate consumption by respiration observed during the batch phase. Then, an exponential increasing feeding regime is activated (after 13 h from the beginning of the experiment) at a Specific Feeding Rate (SFR) value of 0.22 h^–1^, equal to the population specific growth rate, μ. This value is high enough (75% μ_MAX_) to switch back the population to a fermentative metabolism with accumulation of acetate up to 0.3 g l^–1^. Three hours later (16 h from the beginning of the experiment), a constant feeding is applied to the bioreactor beginning at a SFR value of 0.11h^–1^, so that the bacterial cells are allowed to display a respiratory metabolism with no accumulation of acetate. During this phase, however, three glucose pulses are performed inducing temporary acetate production (Figure 2, lower panel).

**FIGURE 3:**
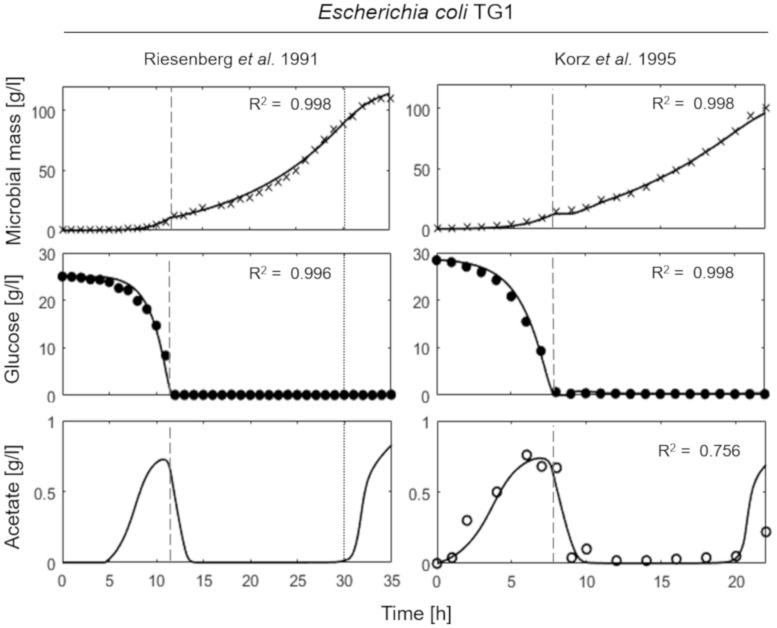
Measured vs. simulated growth of *Escherichia coli* TG1 reproducing Riesenberg et al. (1991) **(left column)** and Korz et al. (1995) **(right column)** experiments. Time series of measured microbial mass (*times symbol*), glucose (*filled circle*) and acetate (*open circle*) data vs. model simulations (*continuous lines*). *Dashed vertical lines* represent the beginning of exponential feeding, while *dotted vertical lines* the beginning of constant feeding.

Figure 3 presents the simulation results of two fed-batch cultures of another *E. coli* strain, namely TG1 (Riesenberg et al., 1991; Korz et al., 1995). The first simulated experiment (Figure 3, left column) is characterized by an initial batch culture lasting 12 h when glucose in the medium is completely depleted, followed by a two-phase fed-batch, carried out at an exponential increasing feeding regime (up to 30 h from the beginning of the experiment) corresponding to a SFR value of 0.11 h^–1^ and then a constant feeding regime (from 30 h onward), starting at 0.11 h^–1^. In the first phase of exponential feeding, the simulated microbial population follows the observed growth corresponding to the feeding regime, while in the second phase (constant feeding) the model properly describes the declining growth rate due to self-produced inhibitory compounds. In turn, the reduced growth rate compared to the glucose feeding induces the metabolic switch reactivating acetate production. In the second simulated experiment (Figure 3, right column) the initial batch phase is followed by a single-phase fed-batch, carried out at a SFR value of 0.17 h^–1^ for the first 3 h and then reduced to 0.14 h^–1^. The simulated dynamics of both experiments are very similar, showing an exponential growth of the cell population in the first fed-batch phase at the imposed μ value. In these conditions, no acetate is produced, since the set-point μ values (0.11 and 0.17 h^–1^ respectively) are below the threshold value for acetate production reported for the strain (Korz et al., 1995).

In brief, in both experiments of Figure 3, a reduction in the growth rate was observed near the end of the run. Such growth reduction appeared when microbial mass achieves a value around 100 g l^–1^, and as explained above is modeled as ascribed to the production of growth-linked inhibitory compounds, different from acetate, consistent with previous findings in yeast (Mazzoleni et al., 2015).

**FIGURE 4:**
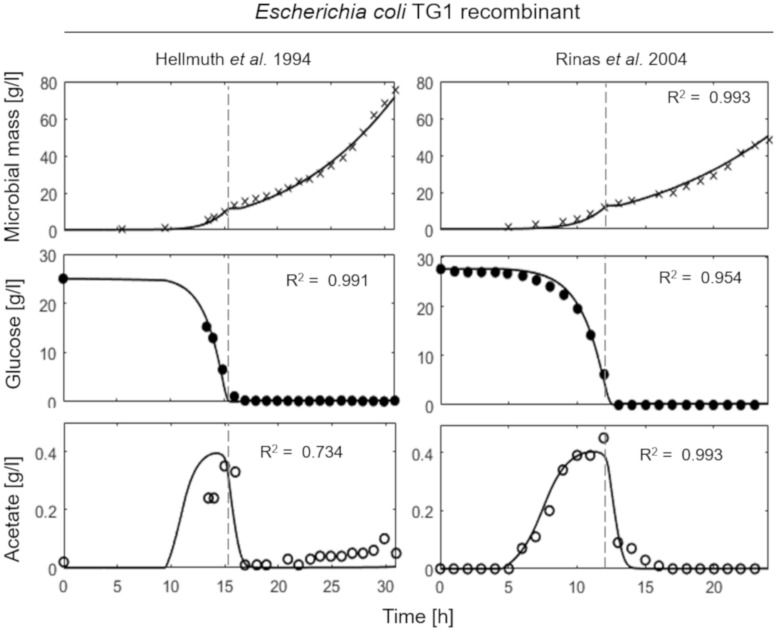
Measured vs. simulated growth of recombinant strains of *Escherichia coli* TG1 reproducing Hellmuth et al. (1994) **(left column)** and Rinas and Hoffmann (2004) **(right column)** experiments. Time series of measured microbial mass (*times symbol*), glucose (*filled circle*), and acetate (*open circle*) data vs. model simulations (*continuous lines*). *Dashed vertical lines* represent the beginning of exponential feeding.

Figure 4 shows the simulations of two fed-batch cultures of recombinant strains of *E. coli* TG1 (Hellmuth et al., 1994; Rinas and Hoffmann, 2004) carried out at SFR of 0.13 and 0.12 h^–1^, respectively. The behavior of the recombinant strains results very similar to the non-recombinant ones of Figure 3, with accumulation of acetate, as indicative of a fermentative metabolism, observed only in the initial batch phase.

**FIGURE 5:**
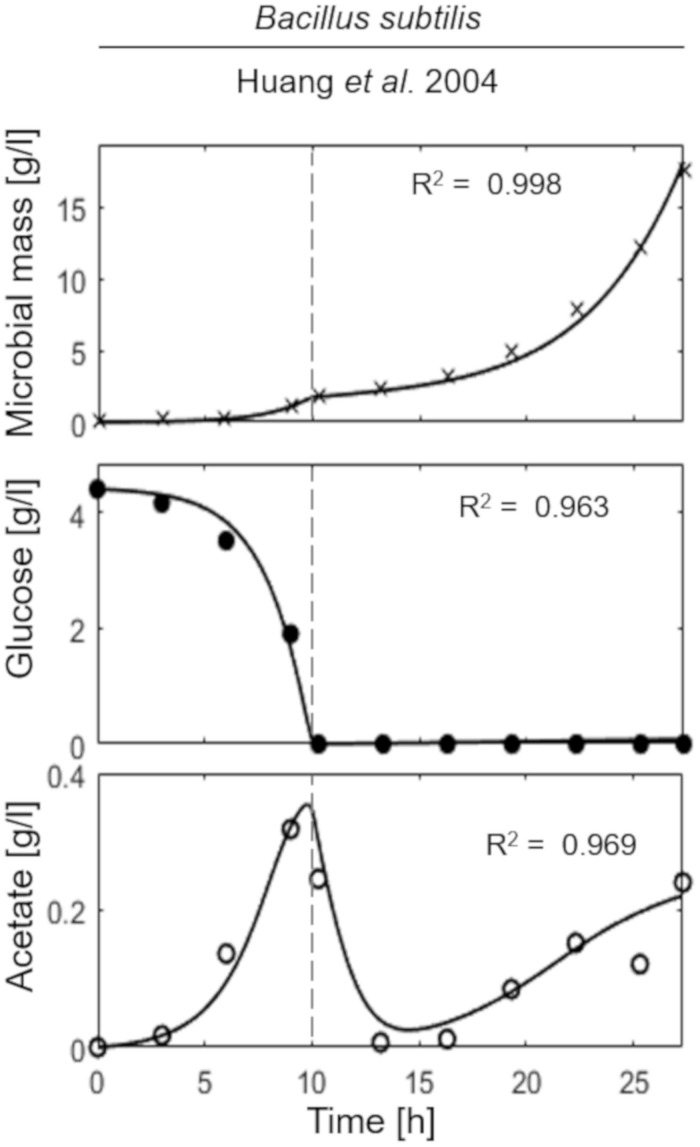
Measured vs. simulated growth of *Bacillus subtilis* reproducing Huang et al. (2004). Time series of measured microbial mass (*times symbol*), glucose (*filled circle*), and acetate (*open circle*) data vs. model simulations (*continuous lines*). *Dashed vertical lines* represent the beginning of exponential feeding, while *dotted vertical lines* the beginning of constant feeding.

**FIGURE 6:**
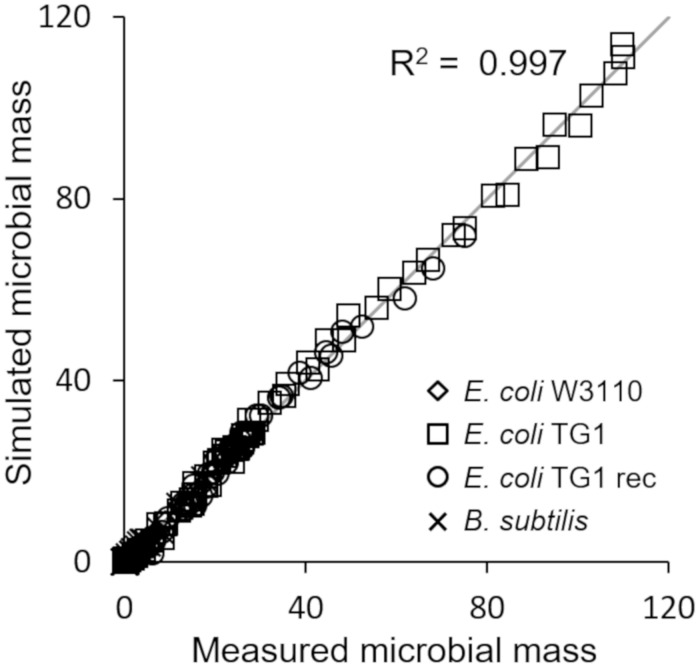
Comparison of measured vs. simulated microbial mass for all presented simulations (Figures 2–5).

**FIGURE 7:**
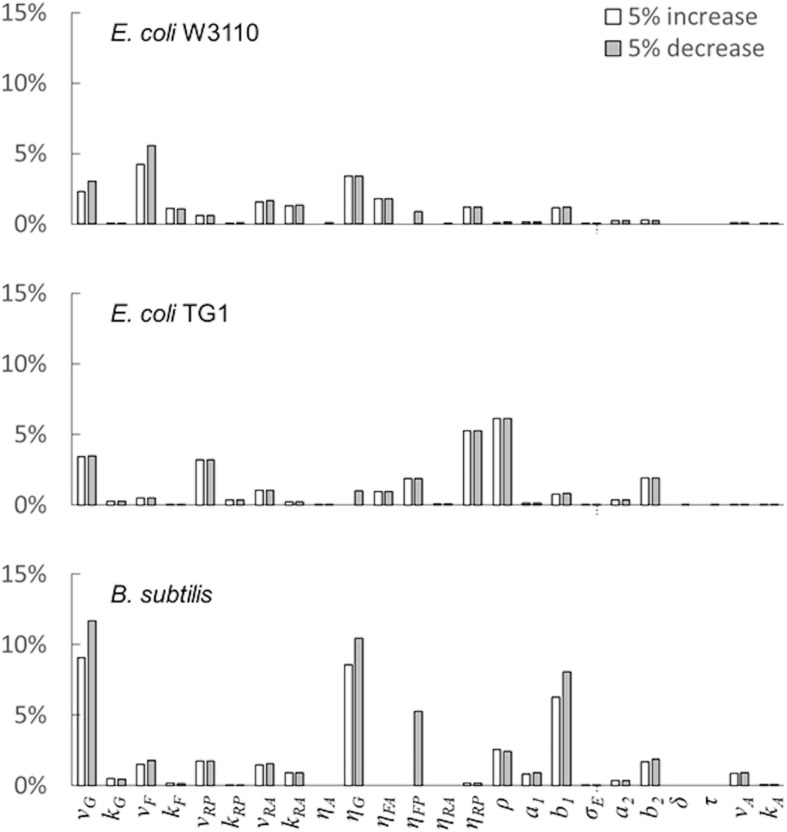
Model parameters’ sensitivity analysis. Variation of the model outcome (compared with baseline simulation) for ± 5% changes in the value of each parameter (see the “Materials and Methods”section for details).

The last simulation presented in Figure 5, shows the growth dynamics of *B. subtilis* cultured in fed-batch carried out at an exponential feeding regime of SFR = 0.12 h^–1^. Also in this case, there is an adequate fit between the model and the experimental data. The culture achieves a very low density (about 17 g l^–1^) at the end of the simulated experiment, and it is characterized by a continuous, although very low, production of acetate. Figure 6 provides a summary of the model simulation performance, showing very good agreement between measured and simulated values of microbial mass for all the selected strains. The results of the sensitivity analysis are presented in Figure 7. The generally low response of the model outcomes to the variation of the parameters shows that the model formulation is robust. In fact, a ± 5% change in each parameter induces significant variations only in a few cases. In particular, two parameters related to glucose uptake (*v*_*G*_ and *η_*G*_*) affected all strains (Figure 7), reflecting the relevance of this process in the model formulation. Differently, it is interesting to notice that each strain showed a specific sensitivity to different parameters. In particular, *E. coli* W3110 appeared to be sensitive to the fermentation process (*v*_*F*_ and *η_*FA*_*); *E. coli* TG1 showed higher responsiveness to the secretion rate of inhibitory compounds (ρ) and the respiration parameters (*v*_*R*_ and *η_*RP*_*); *B. subtilis* showed to specifically respond to the metabolic shift between respiration and fermentation (*b*_1_) and the efficiency of biomass production by fermentation (*η_*FP*_*).

## Discussion

The presented model is capable to reproduce the dynamic behavior of several *Escherichia coli* strains, as well as of *Bacillus subtilis* growing both in batch and fed-batch cultures on glucose as carbon as energy source. The highly significant agreement between experimental data and simulations obtained for different microbial species and strains demonstrates how the process-based System Dynamics approach, already followed in the case of the yeast *Saccharomyces cerevisiae* (Mazzoleni et al., 2015), can be successful to develop a general model of microbial growth in bioreactors, despite the extremely simplified representation of the main physiological functions limited to very few, but fundamental metabolic processes.

Indeed, as for yeasts, also in bacterial cells the dynamic levels of pyruvate, as end metabolic hub of the glycolytic process, play a central role in the control of metabolism. At high glucose concentrations, and consequently at high concentration of glycolytic intermediates, the differential rates of reactions along the fermentative pathway progressively trigger the activation of the overflow metabolism and the consequent repression of respiration (Mazzoleni et al., 2015; de Alteriis et al., 2018).

This essential assumption characterizing the presented model proved indispensable to predict the occurrence of the metabolic shift between respiration and fermentation in two species of prevalent biotechnological interest such as *E. coli* and *B. subtilis*, as shown by the very good agreement between simulations and experimental data (Figure 6). Therefore, acetate is produced in both batch and unrestricted fed-batch cultures, such as the first fed-batch phase of the experiment presented in Figure 2, when the imposed μ value is higher than the critical one for the examined strain. On the contrary, when glucose supply to the bioreactor is controlled, such as in the other simulated experiments of Figures 3, 4, an oxidative metabolism is ensured. Concerning the latter experimental setups of the *E. coli* TG1 strains, the supplied culture medium was the same between recombinant and non-recombinant strains (i.e., mineral medium supplemented with trace elements containing glucose as carbon and energy source). The obtainment of the desired products (beta-galactosidase and human growth factor) by the recombinant strains was achieved by shifting the temperature to 42°C or by addition of 0.5 mM IPTG after 22 h fed-batch phase. In both cases, induction of the products did not affect the dynamics of growth, in terms of glucose consumption and acetate formation, as shown by the simulations.

Noteworthy, in the case of *B. subtilis* (Figure 5), even though the glucose supply was fairly low (SFR = 0.12 h^–1^), a limited production of acetate was observed during all the culture run, showing a less clear-cut metabolic shift for this microbial species. This is also reflected by the high sensitivity of this strain to the parameter related to the metabolic switch (*b*_1_). A further assumption of the proposed model is related to glucose transporters which have been considered as constant within each microbial strain. Clearly, this is a strong simplification since it is known that transporters can be modulated according to glucose availability in the media and future specific studies could address this point more in depth.

Moreover, in the previous *S. cerevisiae* model presented by Mazzoleni et al. (2015), secondary products of fermentation were not considered, being ethanol largely predominant when budding yeast is in conditions of overflow metabolism. Differently, in the case of bacteria, acetate production is followed by a significant production of other partially oxidized products even in aerobic conditions. This is explicitly represented in the model by the description of a mixed fermentation which is assumed to increase with the acetate outflow from the cell. Due to lack of experimental data on such secondary by-products it was impossible to explicitly represent their accumulation in our simulations even though their production is accounted for. The model also considers that the produced acetate can be re-assimilated through the respiratory metabolism at the same time as glucose if both substrates are available in the growth medium, but for simplicity, secondary pathways for acetate consumption were not considered (Anane et al., 2017).

The mechanistic models developed by Pham et al. (1998) to reproduce the growth of *S. cerevisiae* in aerobic fed-batch cultures and later extended to describe the fermentation dynamics of *E. coli* by Xu et al. (1999); Lin et al. (2001), and Neubauer et al. (2003) are probably the most studied microbial macro-kinetic models. Very recently, Anane et al. (2017), refined the previous model formulation to improve the mathematical analyzability and include the latest knowledge on the acetate metabolism of *E. coli*. Our proposed model has a similar level of simplification compared to the abovementioned works, although it was designed to be more general in order to reproduce different microbial species and strains rather than being applied to a single one. Moreover, our model is the only one considering the inhibitory effect on growth and metabolism of self-produced toxic compounds different from fermentation products which is relevant to reproduce the late phases of high cell-density cultures when this phenomenon becomes significant in limiting the growth rate. The phenomenon of self-toxicity regulating cell proliferation is evident only in prolonged fed-batch cultures, and it was clearly pointed out in the case of both wild-type and auxotrophic yeast strains cultured in fed-batch reactors (Mazzoleni et al., 2015). However, in this paper the cell densities achieved in *E. coli* experiments are sufficiently far from the theoretical value of maximum cell density for bacterial cells (200 g d.w. l^–1^) (Lee, 1996). For this reason, the growth decline due to self-toxicity was observed only in the simulated experiment presented in Figure 3, reproducing the experiments of Riesenberg et al. (1991), where the cell density achieved at the end of the run was higher than 100 g d.w. l^–1^. In this case, the final growth decline was clearly visible (Figure 3, left panels) and the model was perfectly capable to reproduce such behavior.

Following a completely different approach, metabolic flux analysis models specifically focused on the central carbon metabolism of *E. coli*, show a detailed description of the metabolic pathways (e.g., Chassagnole et al., 2002; Lemuth et al., 2008). Recently, Millard et al. (2017) developed a detailed kinetic model linking the internal metabolism to the environment and cell proliferation through the description of the dynamics of 62 metabolites, and 68 reactions divided into 3 compartments (environment, periplasm and cytoplasm). This model has been validated using 226 experiments from different sources, allowing the authors to conclude that the self-regulating capabilities of the *E. coli* central metabolism are far more important than expected, also undermining the relevance of gene regulation to explain these dynamics. In typical metabolic flux analyses of Systems Biology, all the measured processes are considered and eventually reduced by selection techniques based on their relevance (bottom-up approach). On this point, Chassagnole et al. (2002) declare: “Because the many biochemical details of the metabolic networks appear overwhelming at first sight, there is a demand for decreasing the enormous complexity of the problem.” As an example, Erdrich et al. (2015) used a combination of “pruning” and “compression” procedures, dramatically reducing the number of reactions from 2384 to 88.

Instead, our modeling procedure directly aims at the identification of a minimal number of processes sufficient to simulate the emergent properties of a complex system (top-down approach) and it is based on logical reasoning on existing knowledge of the system. Then, in this work only those variables relevant for the growth of microbial populations on glucose and their metabolic shift were mathematically described, whereas the many secondary pathways, also directly or indirectly affecting the selected variables, were not taken into account. In summary, our approach is based on the principle of parsimony and has the advantage of keeping the mathematical formulation simple and robust with a reduced number of parameters.

## Conclusion

In conclusion, the results demonstrate how a reductionist System Dynamics approach can be used to formulate simplified macro-kinetic models, still capable to accurately capture the dynamics of biomass growth, glucose consumption and accumulation of fermentation by-products in the medium. The conceptual base of the model, similar to that already proposed for *S. cerevisiae* (Mazzoleni et al., 2015), suggests a unifying theoretical view for all microbial species, with a key role of the metabolic shift phenomenon despite differences in specific molecular mechanisms.

Moreover, the robustness of the results supports a future potential application of the model as a tool for optimization and control of microbial fermentation processes of the main species of biotechnological importance.

## Data Availability Statement

Publicly available datasets were analyzed in this study. This data can be found here: https://doi.org/10.1016/j.bej.2017.05.013, https://doi.org/10.1016/0168-1656(91)90032-q, https://doi.org/10.1016/0168-1656(94)00143-z, https://doi.org/10.1016/0168-1656(94)90215-1, https://doi.org/10.1007/s00449-004-0391-z, and https://doi.org/10.1021/bp034348k.

## Author Contributions

SM, FC, CV, and FG coordinated the model design and its implementation in System Dynamics language. FC, FG, AO, CV, and EP performed the numerical simulations and model calibration. The manuscript was written by FC, EA, and SM. All authors read and approved the final manuscript.

## Conflict of Interest

The authors declare that the research was conducted in the absence of any commercial or financial relationships that could be construed as a potential conflict of interest.
